# Selecting Lentil Accessions for Global Selenium Biofortification

**DOI:** 10.3390/plants6030034

**Published:** 2017-08-26

**Authors:** Dil Thavarajah, Alex Abare, Indika Mapa, Clarice J. Coyne, Pushparajah Thavarajah, Shiv Kumar

**Affiliations:** 1 Plant and Environmental Sciences, 270 Poole Agricultural Center, Clemson University, Clemson, SC 29634, USA; aabare@g.clemson.edu (A.A.); imapapa@g.clemson.edu (I.M.); 2 USDA Agriculture Research Service, Western Regional Plant Introduction Station, Washington State University, Pullman, WA 99164-6434, USA; Clarice.Coyne@ars.usda.gov; 3 BOV Solutions Inc., 1105 Garner Bagnal Blvd, Statesville, NC 28677, USA; rajah.thava@gmail.com; 4 Biodiversity and Integrated Gene Management Program, International Centre for Agricultural Research in the Dry Areas (ICARDA), P.O. Box 6299, Rabat-Institute, Rabat, Morocco; SK.Agrawal@cgiar.org

**Keywords:** lentil, selenium, biofortificaiton, wild germplasm

## Abstract

The biofortification of lentil (*Lens culinaris* Medikus.) has the potential to provide adequate daily selenium (Se) to human diets. The objectives of this study were to (1) determine how low-dose Se fertilizer application at germination affects seedling biomass, antioxidant activity, and Se uptake of 26 cultivated lentil genotypes; and (2) quantify the seed Se concentration of 191 lentil wild accessions grown in Terbol, Lebanon. A germination study was conducted with two Se treatments [0 (control) and 30 kg of Se/ha] with three replicates. A separate field study was conducted in Lebanon for wild accessions without Se fertilizer. Among cultivated lentil accessions, PI533690 and PI533693 showed >100% biomass increase vs. controls. Se addition significantly increased seedling Se uptake, with the greatest uptake (6.2 µg g^−1^) by PI320937 and the least uptake (1.1 µg g^−1^) by W627780. Seed Se concentrations of wild accessions ranged from 0 to 2.5 µg g^−1^; accessions originating from Syria (0–2.5 µg g^−1^) and Turkey (0–2.4 µg g^−1^) had the highest seed Se. Frequency distribution analysis revealed that seed Se for 63% of accessions was between 0.25 and 0.75 µg g^−1^, and thus a single 50 g serving of lentil has the potential to provide adequate dietary Se (20–60% of daily recommended daily allowance). As such, Se application during plant growth for certain lentil genotypes grown in low Se soils may be a sustainable Se biofortification solution to increase seed Se concentration. Incorporating a diverse panel of lentil wild germplasm into Se biofortification programs will increase genetic diversity for effective genetic mapping for increased lentil seed Se nutrition and plant productivity.

## 1. Introduction 

Selenium deficiency is a global public health concern. Recent estimates indicate 15 to 20% of children and adults around the world are Se deficient [1,2]. This means an estimated 30–100 million people are Se deficient, mainly due to low concentrations of bioavailable Se in commonly eaten foods. Biofortification, i.e., enriching staple foods with Se through conventional plant breeding, is considered a sustainable way to increase Se intake and support good general health [3]. Globally, pulses are becoming popular as they are a nutritionally superior, medium-energy food that is low in fat, high in protein, and a good source of micronutrients. For example, a single 50 g serving of lentil (*Lens culinaris* Medikus) provides 3.7–4.5 mg of iron (Fe), 2.2–2.7 mg of zinc (Zn), and 22–34 µg of Se with very low levels of phytic acid (2.5–4.4 mg g^−1^) [4]. Further, lentil is an excellent source of folate [5] and contains a range of low digestible carbohydrates and resistant starch that could modulate human gut microbiome to reduce obesity, overweight, and related non-communicable diseases [6]. 

Selenium is an essential element for mammals but the physiological requirement of Se for higher plants is not well understood [7]. Lower plants, such as algae, require Se for normal growth and development, and green algae contain a Se-dependent antioxidant enzyme, glutathione peroxidase [8]. Lobanov et al. (2009) indicated that sequences for selenoproteins have not been revealed for higher plants, but this conclusion was questioned after a recent study involving de novo assembly and annotation of the complete mitochondrial genome of American cranberry [9]. Specifically, American cranberry was found to contain two copies of tRNA-Sec and a selenocysteine insertion sequence element that were lost in higher plants during evolution [10]. The presence of a selenocysteine insertion in the cranberry mitochondrial genome supports the hypothesis that American cranberry has higher antioxidant activity and high production capability in acidic, low nutrient glacial lake bottom soils with deposits of heavy metals including Se. Although seleno genes were lost during plant evolution, other physiological mechanisms may exist to upregulate plant growth and productivity when grown in Se-rich soils [11]. 

Lentil has a positive response to added low-dose Se fertilizer that varies by genotype. A field study demonstrated that the application of Se increases lentil grain yield, seed Se concentration, and antioxidant levels [12]. Specifically, lentil grain yield and antioxidant responses to added Se varied with genotype, with some cultivars (e.g., CDC Richlea, CDC Viceroy) increasing grain yield and antioxidant activity following Se treatment compared to untreated controls. Consequently, the lentil Se response appears to depend on where the genotype originated, i.e., in low vs. high Se soils [13]. Lentil Se biofortification is possible if based on genotype and location sourcing of diverse lentil germplasm resources including modern cultivated lentil and wild relatives [13,14,15]. Genetic mapping revealed that 352 accessions of cultivated lentil from South Asia and Canada have a narrow genetic diversity with respect to further genetic and breeding enhancement [16]. Therefore, further phenotyping studies with diverse wild accessions will provide greater opportunities to select the most potential breeding lines for Se biofortification efforts. The objectives of this study were to (1) determine the effect of low dose Se fertilizer on seedling biomass, antioxidant activity, and Se uptake of 26 cultivated lentil genotypes from low soil Se regions during germination; and (2) determine the seed Se concentration of 191 lentil wild accessions grown in low Se soils of Terbol, Lebanon.

## 2. Methods and Materials

### 2.1. Materials

Standards, chemicals, and high-purity solvents used for seed digestion, Se analysis, and antioxidant activity were purchased from VWR International, Sigma Aldrich Co. (St. Louis, MO, USA), and Alfa Assar—A Johnson Matthey Company (Ward Hill, MA, USA), and used without further purification. Water, distilled and deionized (ddH_2_O) to a resistance of ≥18.2 MΩ (Milli-Q Water System, Millipore, Milford, MA, USA), was used for sample and reagent preparation. 

### 2.2. Germination Study

Twenty-six cultivated lentil genotypes from the genus Lens subsp. *culinaris* were selected (Table 1). These genotypes were close relatives of high Se uptake lentil cultivars in current production [13,15] and had also been selected for a future genome-wide study based on contrasting physiological response to low dose Se fertilizer. Original lentil seeds were obtained from the USDA-ARS Grain Legume Genetics and Physiology Research Unit, Washington State University, WA, USA, and were multiplied using single plants at Washington State University [17]. Ten surface sterilized seeds from each lentil genotype were placed on sterile petri dishes with sterile absorbent paper, and germinated for five days in clean dark wooden boxes at 22 °C. The treatment design was a complete randomized design with two Se treatments [0 (control; no Se), and 30 kg of Se/ha (6ppm)] with three replicates for each lentil accession, and the entire experiment was repeated twice. Selenium treatment was applied as 4 mL of potassium selenate solution at day 0, 3 and 5 to provide a total dose of 30 kg of Se/ha per petri dish. The control treatment received a similar volume (12 mL for three days) of nano pure water at the same time points. A total of 156 petri dishes were randomly distributed in five similar wooden boxes located in a controlled environment for five days, and then germinating lentil seedlings were transferred to an automated light canopy with day/night temperatures of 22/20 °C, photosynthetically active radiation levels of 200–300 µmol/m^2^/s using a 16-h photoperiod, and 50–60% relative humidity to complete the germination cycle for another two days. On the seventh day, lentils seedlings from each petri dishes were collected, seedling biomass determined (seedling weight), and moisture content of fresh sub-samples measured (via drying at 105 °C for 2 h). The remaining samples were then immediately stored at −40 °C until analysis. Seed Se, antioxidant activity, and biomass data are reported on a dry weight basis (10% moisture).

### 2.3. ICARDA Study

The wild lentil accessions considered in this study originated from 12 different countries and included genus Lens subsp. *culinaris* and five different sub-species (Table 2). A total of 191 lentil wild accessions were grown at the ICARDA field location in Terbol, Lebanon in 2014 using a single row seed per accession. Annual mean precipitation and temperature for 2014 were 247 mm and 8 °C, respectively. Terbol agricultural soils are clay loam with a slightly acidic pH (7.74), 3.24% organic matter, and 103 µg/kg of plant available Se [18]. The Selenium World Atlas indicates Middle Eastern soils are generally low in Se; however, exact soil Se levels were not reported [19]. According to available records, ICARDA fields have been not treated with Se fertilizer, and soil Se data for this field were not available due to logistical issues with importing soils to the USA. At physiological maturity, plants were hand harvested, thoroughly hand cleaned, and then 50 seeds from each of the 191 lentil wild accessions directly shipped to the Pulse Quality and Nutrition Laboratory, Clemson University, SC. Lentil seeds were hand ground using a mortar and pestle and passed through a 0.25 mm sieve prior to measurement of total seed Se concentration. Seed Se data are reported on a dry weight basis (10% moisture). 

### 2.4. Se Analysis

Seed Se concentration was determined using inductively coupled plasma optical emission spectrophotometry (ICP-OES; ICP-6500 Duo, Thermo Fisher Scientific, PA, USA) after nitric acid-hydrogen peroxide digestion [13]. Finely ground seed samples (500 mg) were digested in nitric acid (70% HNO_3_) at 90 °C for 1 h. Samples were then further digested with hydrogen peroxide (30%) before being diluted to 10 mL with nanopure water. Total Se measurements using the ICO-OES method were validated using National Institute of Standards and Technology (NIST) standard reference material 1573a (apple leaves; [Se] = 0.054 ± 0.003 mg kg^−1^). A homogenized laboratory reference material (CDC Redberry: [Se] = 400 ± 100 mg kg^−1^) was also used periodically for quality control. A calibration curve for Se concentration was produced using serial dilutions from 1 to 40 mg L^−1^. The limit of detection for this method was 10 ppt.

### 2.5. Antioxidant Activity

Antioxidant activity of fresh lentil seedlings was measured using the dipheny-picrylhydrazyl (DPPH) free radical scavenging method [12,20]. The DPPH stock solution (1 mM) was prepared by dissolving 19.7 mg DPPH (2, 2-diphenyl-1-picrylhydrazyl) in 50 mL of methanol. A 0.1 mM working solution was obtained by diluting the stock solution (10 mL) with methanol (90 mL). Seedlings were finely ground in liquid nitrogen using a mortar and pestle. One gram of ground seedling was mixed with 3 mL of water, vortexed for 30 s, and then centrifuged at 2000 rpm for 20 min. The supernatant was separated using a 10-mL syringe. Fifty µL of this sample extract were mixed with 3 mL of newly prepared working solution to prepare the sample solutions. A negative control was prepared by mixing 3 mL of 0.1 mM DPPH working solution with 50 µL of distilled water, and a blank control was prepared by mixing 3 mL of methanol with 50 µL of sample extract. All prepared sample solutions were kept in a dark chamber for 30 min at room temperature. After incubation, solutions were centrifuged at 2500 rpm for 10 min. Absorbance was measured at 518 nm and the following formula used to calculate the antioxidant activity (inhibition %):

$$\mathrm{Inhibition}\;\%=\left(\frac{A_{\;\mathrm{Negative}}-A_{\;\mathrm{Sample}}}{A_{\;\mathrm{Negative}}}\right)\times 100.$$

### 2.6. Statistical Analysis

For the germination study, the experiment used a completely randomized design with 26 lentil accessions, three replicates for each accession, and two Se rates (*n* = 156). Data from replicates were combined and data error variances tested for homogeneity. For combined analysis, a mixed model analysis of variance was performed using the PROC GLM procedure of SAS version 9.4 (SAS Institute, Cary, NC, USA) [21], with genotypes and Se rates as the class variables and replicates as a random factor. Means were separated by Fisher’s protected least significant difference (LSD) at *p* < 0.05. For lentil wild accessions, the PROC GCHART procedure of SAS version 9.4 was used for frequency distribution of seed Se concentration for 191 accessions.

## 3. Results

### 3.1. Germination Study 

**Figure 1:**
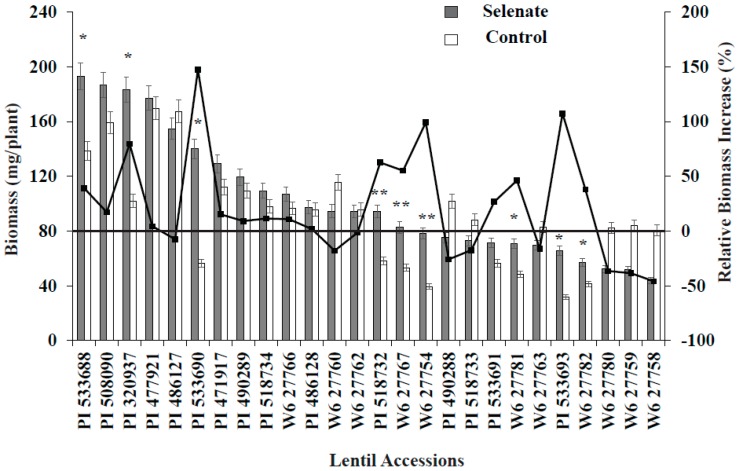
Variation in biomass and relative biomass change for 26 lentil genotypes during germination with added Se fertilizer. *, ** = Significant at *p* < 0.05 and *p* < 0.1, respectively. Relative biomass was calculated based on control biomass weight.

**Figure 2:**
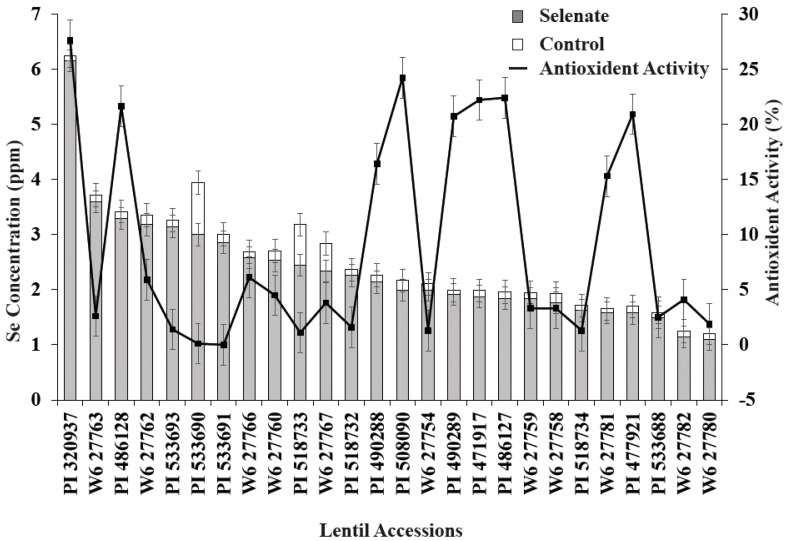
Variation of seedling Se concentration and antioxidant activity for 26 lentil genotypes during germination with added Se fertilizer. Control data are not shown as % inhibition and Se uptake levels were below the detection limit.

Combined statistical analysis of variance showed that lentil genotype and Se treatment significantly (*p* < 0.05) affected seedling biomass, antioxidant activity, and seedling Se concentration (Table 3). The interaction term, genotype × Se treatment, was also significant for all variables. In most cases, Se application increased seedling biomass, seedling antioxidant activity, and seedling Se concentration; however, the magnitude of this effect varied with lentil genotype (Figure 1 and Figure 2). Positive significant correlations were observed between Se treatment and seedling biomass, antioxidant activity, and Se uptake, i.e., Se treatment significantly increased lentil seedling growth, antioxidant activity, and Se nutrition (Table 3). 

Se treatment significantly increased seedling biomass in nine lentil genotypes vs. the control (Figure 1). Similarly, relative biomass increased; specifically, PI320937, PI533690, PI518732, W627767, W627754, and PI533693 demonstrated biomass increases of more than 50% compared to their controls (Figure 1). Among these genotypes, PI533690 and PI533693 demonstrated a >100% biomass increase compared to controls. As expected, Se treatment (30 kg of Se/ha) significantly increased seedling Se concentration for all genotypes; PI320937 showed the highest Se uptake (6.2 µg g^−1^) and W627780 (1.1 µg g^−1^) the lowest (Figure 2). Antioxidant activity for the control treatments was below the detection limit (data not shown). More than 20% antioxidant activity was observed in seven genotypes: PI320937, PI486128, PI508090, PI490289, PI471917, PI486127, and PI477921 (Figure 2). In general, antioxidant activity increased with Se application but responses varied across genotypes. 

### 3.2. ICARDA Study 

**Figure 3:**
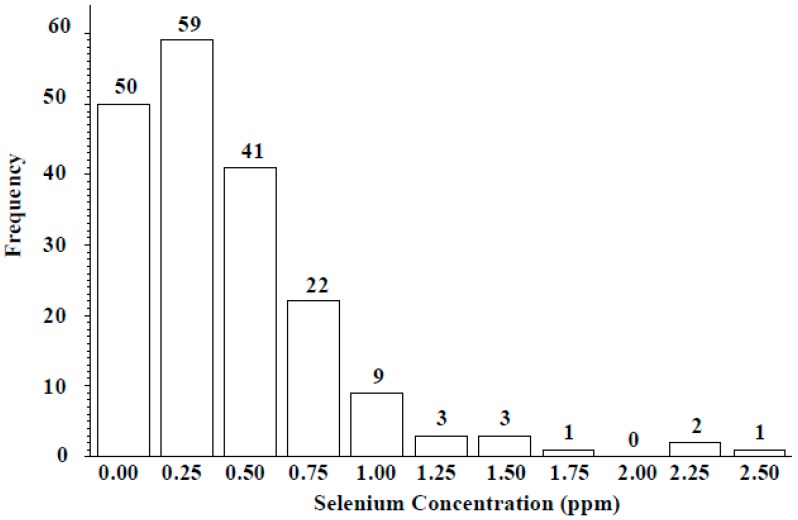
Frequency distribution of seed Se concentration of ICARDA lentil wild accessions grown in Lebanon.

**Figure 4:**
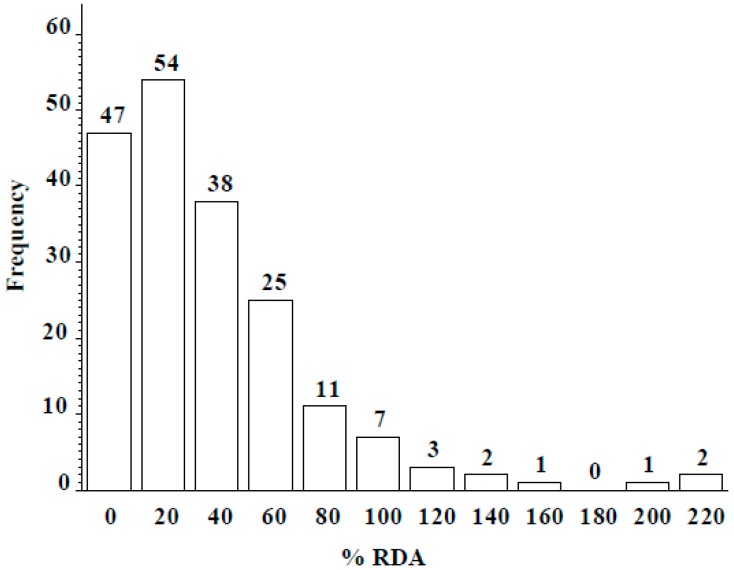
Frequency distribution of percent recommended daily allowance (%RDA: 55 µg/day for an adult) of Se from a 50-g serving of lentil seed from wild accessions grown in Lebanon.

Wild lentil accessions originated from 12 different countries: 100 accessions were from Syria, 57 from Turkey, and the remaining 34 from lentil growing regions including the Middle East, Central Asia, and Europe (Table 4). Across the entire population, seed Se concentration ranged from 0 to 2.45 µg g^−1^, with a mean of 0.41 ± 0.21 µg g^−1^. Seed Se concentrations of wild accessions originating from Syria ranged from 0 to 2.45 µg g^−1^ with a mean value of 0.38 µg g^−1^ and from Turkey ranged from 0 to 2.36 µg g^−1^ with a mean value of 0.54 µg g^−1^ (Table 4). The frequency distribution analysis indicated seed Se concentration for most accessions fell into the 0.25–0.75 µg g^−1^ range (Figure 3). Most wild accessions (144 of 191) would provide an adequate amount of Se (notable % of recommended daily allowance) from a 50-g serving of lentil (Figure 4). For example, 54 accessions would provide at least 20% of the recommended daily allowance of Se and 16 accessions would provide more than 100%. Following 16 lines would provide more than 100% of the recommended daily allowance (RDA): 72868, 116047, 116048, 116052, 119390, 135385, 72527, 72617, 72619, 72628, 72726, 116027, 116028, 116029, 116034, and 114416. Above wild accessions with 1–2 µg Se g^−1^ can be used in lentil breeding programs as a source of high Se content for developing high Se lentil cultivars. 

## 4. Discussion

Selenium is an essential element for the general well-being of humans; however, Se is not an essential element for higher plant survival. Recent literature demonstrates that Se fertilization of food crops increases crop yield, antioxidant protection, drought tolerance, and ultimate Se nutritional quality [12,13,22,23,24]. Literature on the role of Se fertilization during germination on lentil seedling performance has not been reported, however. Results from the current study confirm that the application of Se during lentil germination significantly increases seedling biomass growth, antioxidant activity, plant health, and Se uptake, but these growth responses depend on the genotype. Selenium fertilization effects during the seedling stage may be a genetically driven function in lentil. 

Seedling biomass responses to added Se varied with lentil genotype. Lentil accessions PI533690 and PI533693 showed >100% relative biomass increase while PI320937 and W6-27754 showed >50% relative biomass increase vs. their respective controls (Figure 1). PI320937 showed the greatest response in terms of increased antioxidant activity and seedling Se uptake (Figure 2). These findings challenge the current thinking that Se is not essential for higher plants, and provide a strong rationale to apply Se for improved plant growth and productivity. 

The origin of lentil accessions can be used to explain Se growth responses during germination. Lentil is one of the oldest domesticated pulse crops, originating in the Mediterranean region in the Bronze Age where most soils are low in Se [1]. Our previous studies clearly support the fact that current lentil cultivars in production (e.g., Eston, ILL505, CDC Robin) that originated from low-Se soil countries show the greatest responses in terms of grain yield, seed Se concentration, and speciation to Se fertilization [12,15]. Se fertilization is therefore required to maximize the yield potential of these varieties when grown in low-Se soils. Similarly, data from the current study support the notion that adding low dose Se fertilizer at germination may also activate an initial Se growth response at the seedling stage, leading to healthier plants, increased grain yield, and improved nutritional quality. 

Success in lentil Se biofortification efforts is a function of broad genetic diversity, heritability, and selection abilities. The Se uptake capability of lentil, especially when grown in naturally low-Se soils, could be an important trait and therefore discovering useful genes or alleles for Se uptake is of interest. This paper is the first to our knowledge to report the natural diversity of seed Se levels for the largest wild lentil accession collection. Seed Se concentration varied with *Lens* species and country of origin. Seed Se concentration ranged from 0 to 2.45 µg g^−1^ for accessions originating from Syria, 0 to 2.35 µg g^−1^ for accessions from Turkey, and 0 to 0.91 µg g^−1^ for the other accessions (Table 4). Moreover, frequency distribution analysis clearly indicated approximately 59 wild accessions with 0.25 µg g^−1^ (low seed Se), 41 wild accessions with 0.5 µg g^−1^ (moderate seed Se), 22 accessions with 0.75 µg g^−1^ (high seed Se), and nine accessions with 1.00 µg g^−1^ (very high seed Se) of Se in their seed (Figure 3). Results from studies using molecular markers for lentil breeding efforts might have been limited due to the low genetic diversity among cultivated lentil species [14,16]. However, future genetic Se biofortification is possible by properly identifying genes or alleles responsible for Se uptake by using a larger and more diverse germplasm collection that includes wild accessions. 

Lentil appears to be naturally biofortified with Se when grown in high-Se soils or when fertilized with Se when grown in Se-deficient soils; the latter approach would serve to increase both dietary Se and global lentil production. To address Se deficiency throughout the world, the biofortification of staple food crops with bioavailable Se is required. Several recent studies show Se fertilization may have beneficial effects on staple food crops [15,24,25]. Further, selenomethionine as the dominant Se-species in most food crops suggests that Se is likely to have a good biological availability [13,15,26]. Allaway et al. (1966) showed that Se fertilization to Se-deficient Oregon, USA soils increased the Se concentration of alfalfa from 0.01–0.04 mg kg^−1^ to 2.6–2.7 mg kg^−1^. Since then, the efficacy of Se-fertilization has been well documented around the world [1,25,27]. The efficacy of Se fertilization has been shown for many crops including barley, red clover, perennial ryegrass, and wheat [22,23,24,28]. 

Lentil Se biofortification can be achieved through conventional plant breeding and Se fertilization. Current research suggests that lentil grown in Canada and USA is naturally Se enriched, and fulfills daily dietary Se requirements when consumed in modest amounts (50–100 g d^−1^) [13,25]. Recent biofortification efforts for other metals such as iron and zinc have only focused on plant breeding and biotechnology efforts due to their homeostatic nature. In contrast, Se uptake and biotransformation are controlled by plant physiological conditions and soil Se availability, although the exact metabolic role of Se in higher plants is yet to be determined. Incorporating diverse lentil accessions into a proper lentil mapping population may locate the genes or alleles responsible for Se uptake in lentil. 

## 5. Summary

Lentil is an important food legume in many parts of the world. Exported lentil is currently produced in regions where the soil is relatively rich in Se. However, most regions where lentil is produced and consumed have low soil Se and lack the means to improve seed Se and total yields. Results from this study support the notion that the application of Se at germination not only increases lentil seedling Se uptake but also significantly increases plant productivity in terms of biomass for some genotypes. Incorporating a diverse group of wild accessions into global lentil Se biofortification programs will be of immense benefit as wild accession germplasm has greater phenotypic variation and will thus support effective genetic mapping studies. 

## Figures and Tables

**Table 1:** *Lens culinaris* subsp. *Culinaris* genotypes used in the germination study.

Origin/Source	n	Genotype (Plant Name)
Brazil	3	PI 518732 (CNPH 84–122)
		PI 518733 (CNPH 84–123)
		PI 518734 (CNPH 84–125)
Canada	1	PI 471917 (Eston)
France	3	PI 486128 (Dupuy)
		PI 490288 (Anicia)
		PI 490289 (Mariette)
Germany	1	PI 320937 (ILL505)
Spain	4	PI 533688 (870523-13)
		PI 533690 (Pardina)
		PI 533691 (Lenteja Verdina)
		PI 533693 (Verdina)
USA	14	PI 477921 (Red Chief), PI 486127 (unknown), PI 508090 (Brewer)
		W6 27754 (Parent of 1048-8R), W6 27758 (Parent of CDC Robin)
		W6 27759 (Parent of Eston), W6 27760 (Parent of Giza-9)
		W6 27762 (Parent of ILL 4605), W6 27763 (Parent of ILL 5588)
		W6 27766 (Parent of ILL 7537), W6 27767 (Parent of ILL 8006 BM4)
		W6 27780 (Parent of Milestone), W6 27781 (Parent of Pardina),
		W6 27782 (Parent of Pennell)
Total	26	

**Table 2:** Origin, species/subspecies and accession numbers of 191 lentil wild accessions used in the ICARDA study.

Origin	Species/Subspecies	n	Accession Number
Armenia	*L. culinaris* subsp *orientalis*	1	126939
Cyprus	*L. culinaris* subsp *orientalis*	2	72849, 72595
Czech Republic	*L. culinaris* subsp *unknown*	1	136657
Iran	*L. culinaris* subsp *unknown*	2	72593, 72594
Jordan	*L. culinaris* subsp *orientalis*	5	72847, 72848, 72858, 72864, 72865
Lebanon	*L. culinaris* subsp *odemensis*	1	110846
	*L. culinaris* subsp *unknown*	2	72925, 110824
Poland	*L. culinaris* subsp *orientalis*	1	72600
	*L. culinaris* subsp *unknown*	5	72597, 72598, 136652, 136653, 136658
Syria	*L. culinaris* subsp *odemensis*	15	72640, 72648, 72690, 72697, 72703, 72704 72706, 72758, 72759, 72760, 72893, 107449 119390, 126219, 126220
	*L. culinaris* subsp *orientalis*	49	72534, 72638, 72639, 72645, 72647, 72680 72685, 72688, 72689, 72691, 72699, 72715 72719, 72720, 72750, 72751, 72754, 72761 72765, 72767, 72777, 72778, 72824, 72825 72853, 72854, 72866, 72868, 72869, 72872 72878, 72880, 72881, 72882, 72883, 72884 72887, 72888, 72890, 72892, 107448, 116048 116049, 116052, 126223, 135399, 135443 136777, 139285
	*L. culinaris* subsp *tomentosus*	4	72686, 72820, 72845, 136814
	*L. culinaris* subsp *unknown*	32	72643, 72644, 72646, 72666, 72668, 72669 72672, 72675, 72676, 72692, 72693, 72721 72769, 72770, 72818, 72852, 72870, 72871 72873, 72876, 72877, 107447, 110594, 110803 116043, 116045, 116047, 126221, 126222 135385, 135410, 135415
Tajikistan	*L. culinaris* subsp *odemensis*	1	72899
	*L. culinaris* subsp *orientalis*	5	72904, 72905, 72907, 136679, 140379
Turkmenia	*L. culinaris* subsp *orientalis*	1	72901
Turkey	*L. culinaris* subsp *odemensis*	2	72562, 136673
	*L. culinaris* subsp *orientalis*	33	72527, 72529, 72530, 72602, 72604, 72606 72608, 72610, 72611, 72612, 72613, 72616 72617, 72618, 72619, 72620, 72621, 72626 72627, 72628, 72629, 72632, 72726, 72746 72748, 72816, 72830, 114416, 116008, 116010 116029, 136677, 72623
	*L. culinaris* subsp *tomentosus*	1	72625
	*L. culinaris* subsp *unknown*	21	72724, 72742, 72743, 72744, 72800, 72801 72804, 72805, 72831, 72835, 72836, 72855 116015, 116027, 116028, 116034, 136662 136665, 136666, 136669, 136670
Uzbekistan	*L. culinaris* subsp *odemensis*	1	72900
	*L. culinaris* subsp *orientalis*	5	72895, 72896, 72897, 72908, 72909
	*L. culinaris* subsp *unknown*	1	72592
	Total	191	

**Table 3:** Combined analysis of variance for 26 lentil genotypes during germination with response to Se fertilizer.

Source	df	Mean Squares		
		Biomass	Antioxidant	Se Uptake
Genotype	25	*	*	*
Se treatment	1	*	*	*
Replication	2	NS	NS	NS
Genotype × Se treatment	25	*	*	*
Error	102	0.5	3.1	0.1
Person correlation coefficient (n = 156)				
Biomass		1.00	0.41 *	0.20 *
Antioxidant		0.41 *	1.00	0.54 *
Se uptake		0.20 *	0.54 *	1.00

df, degree of freedom; * *p* < 0.05; NS, not significant. Values indicate Pearson correlation coefficients (*r*). Bold values indicate significant correlation. ** *p* < 0.05.

**Table 4:** Seed Se concentration of 191 lentil wild accessions grown in Lebanon.

Country of Origin	No of Samples	Seed Se Concentration (µg g^−1^)	
		Range	Mean
Armenia	1	0.59	0.59
Cyprus	2	0.20–0.51	0.36
Czech Republic	1	0.91	0.91
Iran	2	0.30–0.90	0.60
Jordan	5	0.18–0.81	0.34
Lebanon	3	0.02–0.49	0.23
Poland	6	0.01–0.66	0.32
Syria	100	0.00–2.45	0.38
Tajikistan	6	0.00–0.53	0.16
Turkmenia	1	0.31	0.31
Turkey	57	0.0–2.36	0.54
Uzbekistan	7	0.0–0.76	0.18
Mean ± SD			0.41 ± 0.22
Total	191		

SD, Standard deviation (*n* = 191).
